# Synchronous gallbladder metastasis originating from residual gastric cancer: a case report and the review of literature

**DOI:** 10.1186/s40792-022-01442-5

**Published:** 2022-05-07

**Authors:** Ami Kawamoto, Koichi Kimura, Kosuke Hirose, Takuma Izumi, Daisuke Taniguchi, Hiroko Yano, Yuichiro Kajiwara, Ryosuke Minagawa, Kazuhito Minami, Yumi Oshiro, Takashi Nishizaki

**Affiliations:** 1 Department of Surgery, Matsuyama Red Cross Hospital, 1, Bunkyo-cho, Matsuyama, Ehime 790-8524 Japan; 2 Department of Pathology, Matsuyama Red Cross Hospital, 1, Bunkyo-cho, Matsuyama, Ehime 790-8524 Japan

**Keywords:** Residual gastric cancer, Metastasis, Signet-ring cell carcinoma, Gallbladder

## Abstract

**Background:**

Gastric cancer rarely metastasizes to the gallbladder. Furthermore, there has never been a case report of simultaneous gallbladder metastasis from residual gastric cancer. Here, we report a case of synchronous gallbladder metastasis originating from a residual gastric cancer.

**Case presentation:**

A 67-year-old man underwent a follow-up upper endoscopy 18 months after a partial gastrectomy for gastric cancer; an ulcerative lesion was found in the remnant stomach at the gastrojejunal anastomosis. A biopsy revealed gastric signet-ring cell carcinoma (SRCC). A full-body examination revealed no abnormalities other than gallstones in the gallbladder. With a diagnosis of residual gastric cancer (cT2N0M0 cStage I), the patient underwent open total gastrectomy and cholecystectomy. Macroscopic findings of the resected specimen revealed thickening of the gallbladder wall; however, no obvious neoplastic lesions were found on the mucosal surface of the gallbladder. The pathological findings showed that the SRCC had invaded the submucosa of the gastrojejunostomy site with a high degree of lymphatic invasion and lymph node metastases. SRCCs were also found in the lymphatic vessels of the gallbladder wall. These findings suggested the possibility of gallbladder metastasis through lymphatic vessels. The patient and his family members refused postoperative chemotherapy. Ten months after the operation, the patient experienced respiratory failure due to lymphangitis carcinomatosa and died.

**Conclusions:**

At present, it is difficult to determine whether resection of the gallbladder contributes to an improved prognosis of gastric cancer patients. However, reports in such cases demonstrate that gallbladder metastasis could be a poor predictor of prognosis for gastric cancer.

## Background

Gallbladder metastasis from gastric cancer is extremely rare [1]. A previous report showed that the occurrence rate of gallbladder metastasis from gastric cancer is only 0.06% [2]. In addition, the preoperative diagnosis of gallbladder metastasis from gastric cancer is difficult due to few reported cases and a lack of specific imaging findings. Furthermore, imaging findings may also overlap with features of other gallbladder diseases, such as cholecystitis, gallbladder polyps, and adenomyomatosis of the gallbladder [3].

The prognosis of gallbladder metastasis is poor. Yoon et al. [4] reported that the median survival time for metastatic gallbladder tumors 8.7 months. Here, we present a case of synchronous gallbladder metastasis originating from a residual gastric SRCC in addition to a review of the literature.

## Case presentation

**Fig. 1:**
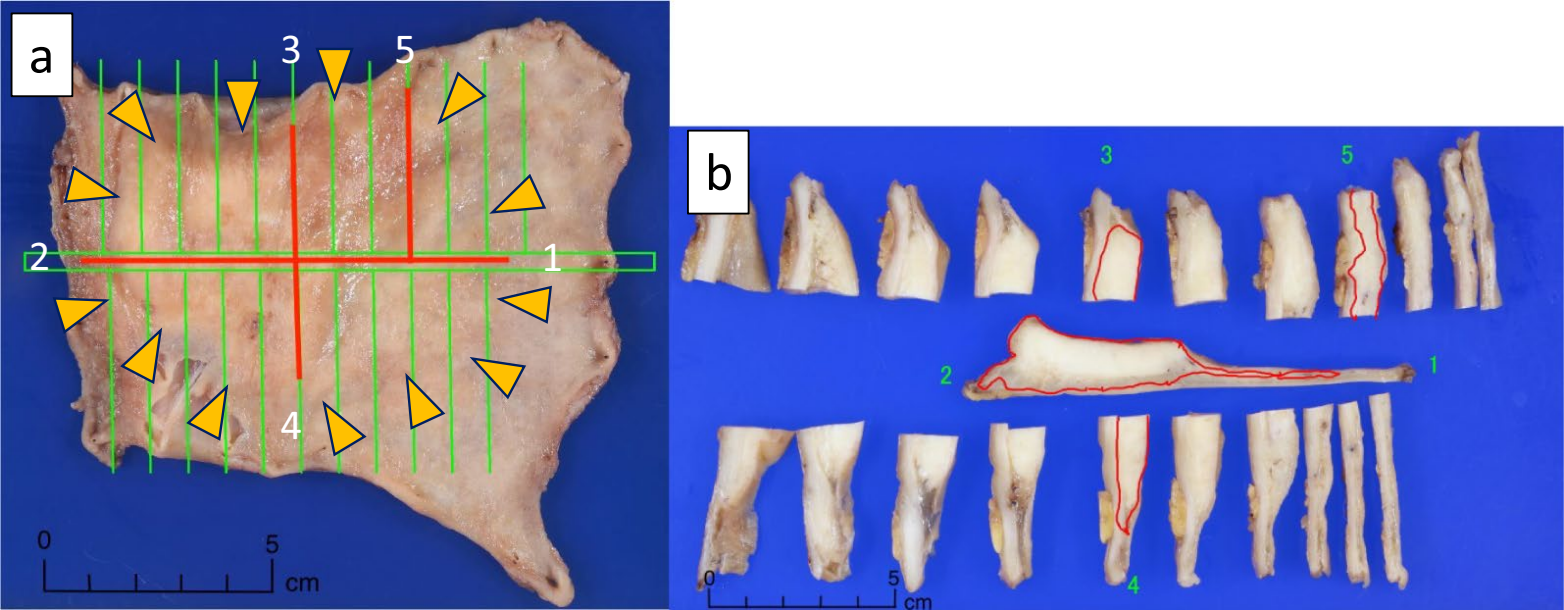
**a** Macroscopic findings of the resected gastric lesion specimen at the time of the first surgery(1: proximal site, 2: distal site). The green line is the dividing line at the time of pathology specimen preparation. Poorly differentiated adenocarcinoma was identified in the area of the red line. Together with the macroscopic view, the area indicated by the arrow is presumed to be the tumor. **b** The lesion is located in the area indicated by the red line. Histologically, the lesion was extensively found along the submucosal layer (red line). The margins were negative

A 67-year-old man was diagnosed with gastric cancer via an upper endoscopy for the evaluation of esophageal varices while commuting to our hospital for alcoholic cirrhosis (Child–Pugh classification: grade B). The patient tested positive for *Helicobacter pylori* infection. He had a history of drinking > 108 g of alcohol per day for 40 years (stopped 5 years ago) and smoking 40 cigarettes per day for 40 years (stopped 8 years ago). Due to impaired liver function and the presence of esophageal varices, the surgical risk was high, and partial gastrectomy (D0 dissection, with antecolic Roux-en-Y reconstruction) was performed with preservation of the left gastric artery. The postoperative pathological diagnosis was LMD, Gre, Type 3, 90 × 55 mm, poorly differentiated adenocarcinoma, non-solid type, pT2 (MP), INFc, Ly0, V0, pPM0 (15 mm), pDM0 (3 mm), and pN0 (#3:0/9, #4d:0/2) according to the Japanese Classification of Gastric Carcinoma [5] and T2N0 Stage IB in accordance with the 8th edition of the Union for International Cancer Control classification[6], with no signet-ring cell (Fig. 1).

**Fig. 2:**
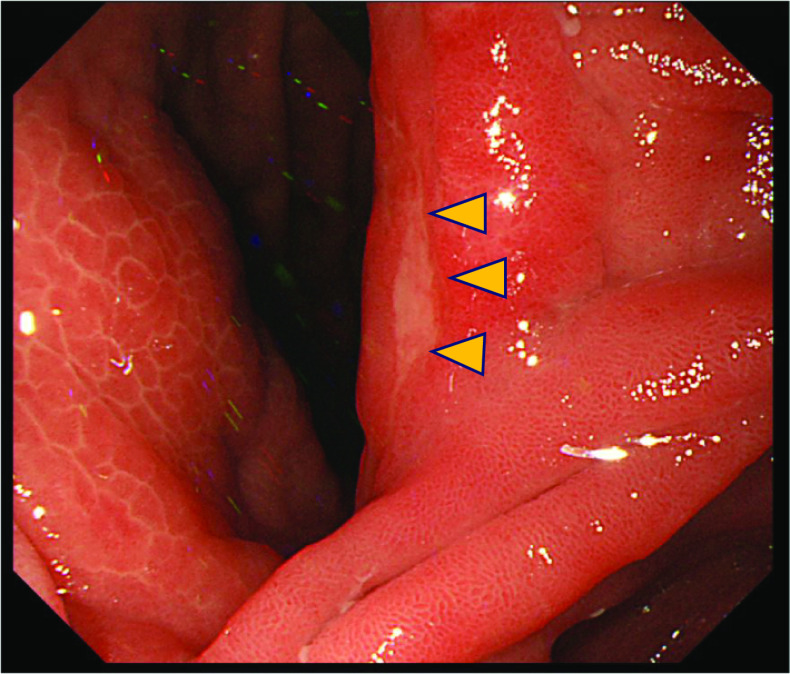
Endoscopic findings of the gastrojejunal anastomosis. Endoscopic examination revealed 0–IIc. A biopsy specimen obtained from the tumor shows signet-ring cell carcinoma (SRCC) (arrow)

The patient underwent a follow-up upper endoscopy 18 months after gastric cancer surgery. A 0–IIc lesion was found at the gastrojejunal anastomosis; a biopsy of the same site revealed SRCC (Fig. 2). Blood chemistry tests showed that liver function improved with abstinence (Child–Pugh classification: grade A), and other blood count and biochemistry parameter levels were within normal ranges. Levels of tumor markers, carcinoembryonic antigen (2.2 ng/ml), and carbohydrate antigen 19–9 (11.6 U/ml) were within normal limits.

**Fig. 3:**
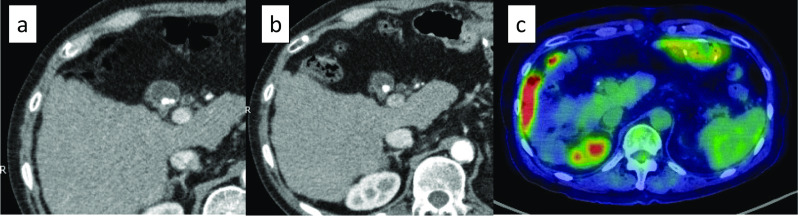
**a** CT findings 2 years before the second gastric surgery. **b** Preoperative CT findings. It was difficult to identify the primary site, and there were no findings suggestive of distant or LN metastasis. There were no abnormal findings in the gallbladder, except for the presence of gallstones, and there were no changes over time. **c** FDG-PET/CT showing negative uptake in the stomach, LNs, and gallbladder

Abdominal computed tomography (CT) showed difficulty in identifying the primary lesion, and there were no findings suggestive of lymph node (LN) metastasis or distant metastasis. There were no abnormal findings in the gallbladder wall, except for the presence of gallbladder stones, with no changes observed for 2 years (Fig. 3a, b). Fluoro-deoxyglucose (FDG)-positron emission tomography (PET)/CT revealed negative uptake in the gastric lesion (Fig. 3c).

Surgical findings showed no distant metastasis, such as peritoneal dissemination or liver metastasis, or obvious LN metastasis. In addition, the intraoperative washing cytology was negative. There was also no obvious exposure of the serous surface to residual gastric cancer. In the gallbladder, the wall of the body appeared thickened; however, there were no obvious neoplastic lesions. Therefore, we performed a total gastrectomy with LN dissection and cholecystectomy to prevent cholecystitis.

**Fig. 4:**
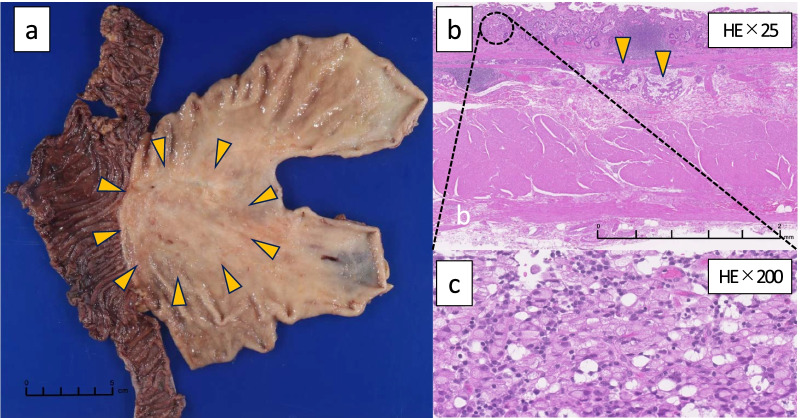
** a** Macroscopic findings of the resected specimen of the gastric lesion was a type 5 tumor based on the Borrmann classification, 73 × 55 mm, and was located in the gastrojejunal anastomosis (arrow). **b** Histological findings of gastric cancer showing invasion of cancer cells into the layer of tissue under the mucosa, and masses of cancer cells in the lymphatic vessels (× 25, hematoxylin and eosin (HE) stain, arrow). **c** Signet-ring cell carcinoma (SRCC) and poorly differentiated carcinoma were observed in the mucosa of the stomach (× 200, HE stain)

As for the macroscopic findings, a thickening of the mucosa on the gastric side of the previous gastrojejunostomy anastomosis from the previous operation was observed, and the main lesion was 75 × 55 mm (Fig. 4a). Histopathologically, the stomach showed an SRCC and a non-seminomatous poorly differentiated adenocarcinoma in the mucosa (Fig. 4b, c). The cancer cells showed strong lymphatic invasion and had invaded the layer of tissue under the serosa. The LNs showed metastasis in No. 1, 3, and 4sa (11/14).

**Fig. 5:**
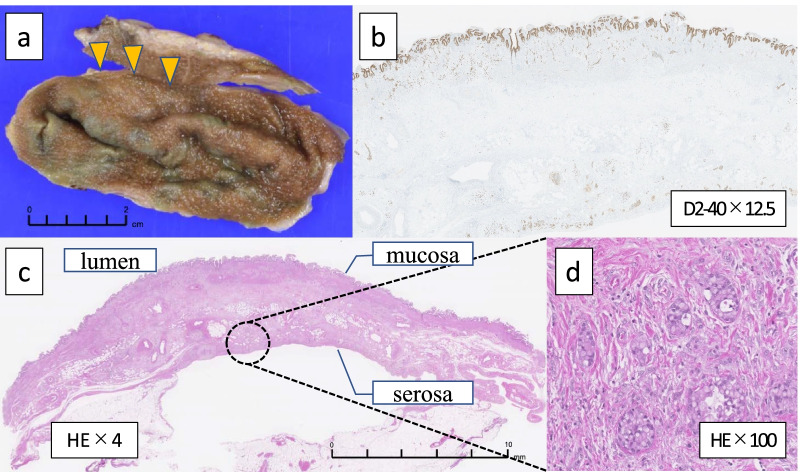
**a** Macroscopic finding of the resected specimen of the gallbladder showed a partial wall thickening suggestive of gallbladder adenomyosis (arrow). **b**–**d** Histological specimen of the gallbladder showing no mucosal epithelial lesions. The lymphatic vessels of the gallbladder wall were extensively filled with tumor cells, which was especially evident in the grossly thickened areas (**b** × 12.5, D2-40 stain; **c** × 4, HE stain; **d** × 100, HE stain)

The gallbladder showed partial wall thickening, suggestive of adenomyosis (Fig. 5a). Histological examination of the gallbladder revealed no mucosal epithelial lesions. The lymphatic vessels of the gallbladder wall were extensively filled with tumor cells, especially in the grossly thickened areas (Fig. 5b–d). Since the histological findings of the gastric and gallbladder lesions were identical, the gallbladder lesion was diagnosed as gallbladder metastasis of the gastric cancer. The final pathological stage was T3N3aM1 (GAL) stage IV.

The patient was discharged on the 11th postoperative day in a good condition. The patient and his family members refused postoperative chemotherapy. There was no metastasis or recurrence other than lymphangitis carcinomatosa, and the patient died of respiratory failure 10 months postoperatively.

## Discussion

In this case, gallbladder metastasis was incidentally discovered 18 months after residual gastric cancer surgery. Regarding the gastric lesions, the initial lesions had no lymphatic invasion (Ly0) or lymph node metastasis (#3:0/9, #4d:0/2); thus, lymphatic invasion was different from that of the recurrent lesions. SRCCs tend to spread laterally across the normal mucosal surface [7] and gastric cancer is highly heterogeneous [8]. In this case, it is possible that a new type of SRCC may have developed; however, considering that the extent of the tumor was difficult to identify visually, and that the proximal margin of the first surgery was short (15 mm), the possibility that cancer cells may have remained histologically in an area that could not be confirmed macroscopically and that remaining tumor cells with high lymphatic invasion potential had recurred and proliferated was considered.

Gallbladder metastases are rare [1]. Chan et al. [2] reported 36 cases of gallbladder metastasis (0.46%) out of 7,910 excised gallbladders in patients with malignant tumors, of which only five cases (0.06%) originated from gastric cancer. To the best of our knowledge, only 18 cases of gallbladder metastasis from gastric cancer have been reported from 1990 to 2020 in Japan, in addition to our own cases. Furthermore, gallbladder metastasis occurring in residual gastric cancer has not been reported in the literature and is considered extremely rare. Seventeen cases of the available pathological type had poorly differentiated histology, such as por or sig (70%). Eleven of 16 cases (69%) with available pathological data showed lymphatic invasion (Ly ≥ 2) and LN metastasis (N ≥ 2). In addition, macroscopic findings of the gallbladder often include submucosal masses and thickened walls. In only one of 17 cases (6%), gallbladder metastasis was clinically diagnosed preoperatively, indicating that preoperative diagnosis is difficult. In addition, 10 of 19 patients (53%) died within one year of surgery (Table 1).

**Table 1:** Reported cases of gallbladder metastasis from gastric cancer in Japan

Case	Year	Age	Sex	Gastric cancer					Gallbladder		Metastatic pathway	Cause of death	Duration/Prognosis
				Histological type	Ly	V	N	Dissemination	Preoperative diagnosis	Macroscopic findings			
1	1990	44	M	Por	2	0	3	+	GB polyps	SMT	L	Carcinomatous meningitis	8 months/Death
2	1996	54	M	ND	ND	ND	ND	ND	ND	SMT	ND	ND	ND
3	1996	63	F	ND	ND	ND	ND	ND	ND	SMT	ND	ND	ND
4	1999	62	M	Sig	2	2	1	ND	GB stones	–	ND	DIC	4.5 months/Death
5	2002	86	F	Adenosquamous	3	2	1	+	WT	WT	L	Acute renal failure	30 days/Death
6	2006	88	M	Endocrine	3	2	2	−	NA	–	L or H		8 months/Alive
7	2007	54	M	Por	2	1	2	−	GB stones	WT	L	Obstructive jaundice Carcinomatous peritonitis	5 months/Death
8	2008	71	M	Tub2	1	0	2	+	GB stones	–	L	Carcinomatous peritonitis Pneumonia	53 days/Death
9	2008	76	M	Por	2	2	1	−	NA	–	H	Bone metastasis Weakness	43 days/Death
10	2009	54	M	Por	2	1	3	ND	GB stones	SMT	L		3 years/Alive
11	2011	66	F	Por	1	0	1	−	NA	–	L		13 months/Alive
12	2013	50	F	Tub2	3	0	1	−	Primary GB tumor	WT	L		33 months/Alive
13	2014	68	F	Sig	3	1	2	−	Primary GB tumor	WT	L		24 months/Alive
14	2014	62	F	Por	3	2	3	−	NA	SMT	L	Recurrence of cancer	9 months/Death
15	2014	55	M	Tub2	2	1	3	−	GB metastasis	SMT	L or H		ND
16	2019	66	F	Por + tub2	1	1	3	−	GB stones Adenomyomatosis	SMT	L	Lymphangitis carcinomatosa	7 months/Death
17	2020	71	M	Sig	0	+	3	−	GB stones	–	L	Death of cancer	199 days/Death
18	2021	84	M	Por	ND	ND	ND	+	Acute cholecystitis	WT	Peritoneal dissemination		ND/Alive
Present case	2020	67	M	Sig	1	0	3	−	GB stones	WT	L	Lymphangitis carcinomatosa	10 months/Death

*ND* no data, *por* poorly differentiated adenocarcinoma, *sig* signet-ring cell carcinoma, *tub2* moderately differentiated tubular adenocarcinoma, *NA* no abnormality, *GB* gallbladder, *WT* wall thickness, *SMT* submucosal tumor, *L* lymphogenous, *H* hematogenous

Choi et al. [3] classified the CT findings of metastatic gallbladder tumors into wall thickening and protruding lesions into the lumen, and identified specific growth patterns based on the type of primary tumor. They stated that metastatic gallbladder adenocarcinoma was often observed to have invasive wall thickening lesions, while the polyp type was mainly associated with non-adenocarcinomatous histological types. However, these findings have also been observed in primary gallbladder tumors. In addition, the findings of gallbladder metastasis are diverse, and the findings on CT may overlap with those of other gallbladder diseases [9], thus preoperative diagnosis is difficult.

Regarding the route of metastasis of gastric cancer to the gallbladder, according to previous reports, the presence of advanced LN metastasis and lymphatic vessel invasion of tumor cells caused obstruction of various lymphatic vessels and the development of abnormal lymphatic pathways, leading to lymphatic metastasis to the stroma within the gallbladder wall [10]. The lymphatic flow from residual gastric cancer, such as in our case, develops a different flow from that of the normal stomach depending on the initial surgical technique, degree of LN dissection, and degree of adhesions after surgery [11]. In residual gastric cancer after pyloric gastrectomy without dissection of the left gastric artery, lymphatic flow in the upper part of the gastric body is reported to follow the following pathways: from the cardia, right side of the gastric fornix, and lesser curvature of the gastric body to the left gastric artery; from the left side of the gastric fornix and greater curvature of the gastric body to the left gastroepiploic artery and short gastric artery to the splenic artery; from the gastric fornix and posterior wall of the gastric body to the splenic artery via the posterior gastric artery; and from the cardia to the left diaphragmatic artery [11]. The lymphatic flow at the anastomosis depends on the reconstruction method, and it is thought that the adhesion of the remaining stomach to the surrounding tissues leads to the formation of new lymphatic channels, which can easily metastasize to mesenteric LNs adjacent to the anastomosis and the root of the superior mesenteric vein (No. 14) in cases of residual gastric jejunal anastomosis such as Roux-en-Y, which is high in cancer [12].

**Fig. 6:**
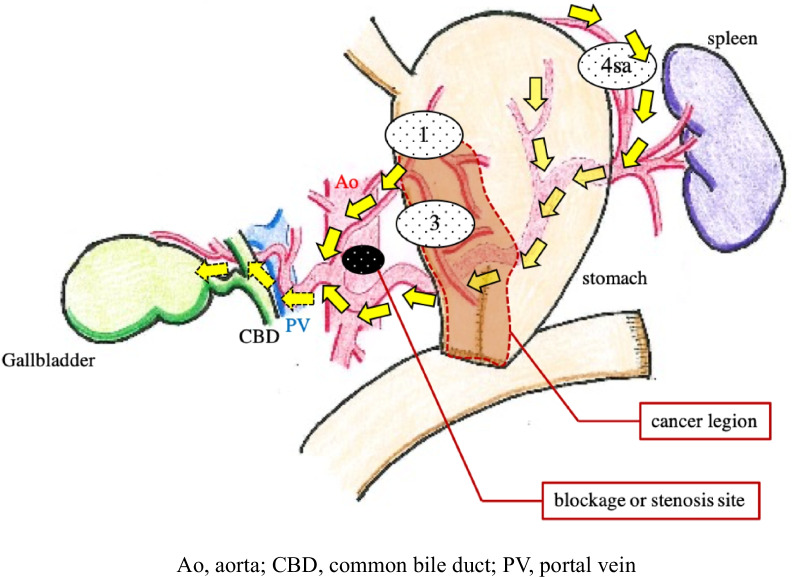
The speculated pathway of metastasis from gastric cancer to gallbladder. The black circled area is blockage or stenosis site in the lymphatic vessels. Imaginary abnormal lymphatic backflow shown with the dotted pathway

In our case, although the gastric lesion showed invasion into the subserosa, the possibility of disseminated metastasis or direct invasion to the gallbladder was considered to be low. This was because there was no continuity between the gastric and gallbladder lesions, no change in the serous surface of the gallbladder, no peritoneal dissemination, and the intraoperative washing cytology was negative. In addition, in our case, the gastric lesion was negative for venous invasion (V0) and showed severe lymphatic invasion (Ly1) and LN metastasis (N3). The gallbladder lesion also showed a high degree of lymphatic invasion, and the histology was identical to that of the gastric lesion. Gastrojejunostomy cases are said to easily metastasize to the periaortic LN due to the aforementioned characteristics of lymphatic flow [12]. Since there were no grossly obvious tumor lesions in the falciform ligament or other parts of the liver, spleen, and pancreas in this case, extended LN dissection was not performed; therefore, the actual extent of LN metastasis is unknown. However, since metastasis was found in dissected LN 1, 3, and 4sa, it was inferred that cancer cells flowed from the left gastric artery to the celiac artery, and from the left gastroepiploic artery and posterior gastric artery to the celiac artery through the splenic artery. It was speculated that lymphatic vessel obstruction or narrowing in the downstream region caused lymphatic reflux into the common hepatic artery and right hepatic artery, which metastasized lymphatically to the gallbladder (Fig. 6).

The prognosis of patients with gallbladder metastasis from gastric cancer, including our patient, is extremely poor, with a median survival time of 7 months. To establish a treatment strategy in such cases, a detailed case analysis is required.

## Conclusions

In this study, a review of the literature revealed that gallbladder metastasis from gastric cancer has poorly differentiated histology (por/sig) in 70% of cases, lymphatic invasion with Ly ≥ 2, and LN metastasis with N ≥ 2 in 69% of cases. Preoperative diagnosis was difficult; only one of the 17 reported patients had a correct diagnosis before surgery. In addition, 10 of 16 patients died within 1 year after surgery. At present, it is difficult to determine whether resection of the gallbladder contributes to improved gastric cancer prognosis; however, the diagnosis of gallbladder metastasis may be a poor predictor of prognosis for gastric cancer.

## Data Availability

Data sharing is not applicable to this article, as no datasets were generated or analyzed during the current study.

## Abbreviations

SRCC: Signet-ring cell carcinoma
LN: Lymph node
CT: Computed tomography
FDG: Fluoro-deoxy-glucose
PET: Positron emission tomography
